# Comparative transcriptome analysis of *Liriomyza trifolii* (Burgess) and *Liriomyza sativae* (Blanchard) (Diptera: Agromyzidae) in response to rapid cold hardening

**DOI:** 10.1371/journal.pone.0279254

**Published:** 2022-12-15

**Authors:** Junaid Iqbal, Xiao-Xiang Zhang, Ya-Wen Chang, Yu-Zhou Du

**Affiliations:** 1 School of Horticulture and Plant Protection & Institute of Applied Entomology, Yangzhou University, Yangzhou, China; 2 Joint International Research Laboratory of Agriculture and Agri-Product Safety, The Ministry of Education, Yangzhou University, Yangzhou, China; USDA Agricultural Research Service, UNITED STATES

## Abstract

The ability of insets to react efficiently to fluctuation in temperature is crucial for them to survive in variable surroundings. Rapid cold hardening (RCH) is a process that increase cold tolerance in most insect species. The molecular mechanisms of RCH remain largely unknown, and whether it is associated with transcriptional changes is unclear. In this study, we compared the transcriptomes of *Liriomyza trifolii* and *L*. *sativae* exposed to RCH to investigate the transcript abundance due to RCH in both species. RNA-seq revealed 93,166 assembled unigenes, and 34,303 of these were annotated in the *L*. *trifolii* and *L*. *sativae* transcriptome libraries. After a 4-h treatment at 1°C (RCH) compared with control, 268 and 606 unigenes were differentially expressed in *L*. *trifolii* and *L*. *sativae*, respectively. When comparing pupae exposed to 2h cold shock directly with pupae went through 4h acclimation prior to 2h cold shock, 60 and 399 unigenes were differentially expressed in *L trifolii* and *L sativae*, respectively. Genes that were commonly expressed in both *L*. *trifolii* and *L*. *sativae*, included cytochrome P450, cuticular protein, glucose dehydrogenase, solute carrier family 22 and cationic amino acid transporter. Additionally, several pathways including galactose metabolism and peroxisome were significantly enriched during RCH. Our results show that the transcriptional response is correlated with RCH in the pupal stage of the two *Liriomyza* species, but more transcriptional changes were identified in *L sativae* than in *L*. *trifolii*.

## Introduction

Insects are the most diverse animals on the planet and have vital roles in ecosystems [1]. Their life cycle can be directly affected by several abiotic factors including light, humidity and temperature, and these factors affect their distribution and abundance [2]. Insects are ectotherms and therefore rely on the environmental heat sources. During the winter, low temperatures can pose a threat to the persistence of insect populations [3–5]. Insect development occurs within a specific temperature range, so changes in temperature will influence development, life cycle and survival. To cope with low temperature, insects have evolved various mechanisms [6, 7]. Cold acclimation is a process that increases cold tolerance and allows insects to remain active for longer period of time during seasonal changes [8]. Cold tolerance can be achieved through long-term cold acclimation, where insects undergo natural and gradual temperature changes [9]. Besides long-term acclimation, cold tolerance can also be achieved by a short-time exposure (e.g. 30 mins) to a mild cold temperature, known as rapid cold hardening (RCH) [10], which may benefit overwintering insects when they are exposed to a sudden decrease in temperature for a short period in the spring and autumn months [11]. RCH improves cold resistance in a variety of insect species, which is critical for their survival [11, 12]. In addition to enhancing cold survival, RCH serves as protection against non-lethal cold injury because it preserves essential functions such as locomotion, reproduction, and energy balance (Reviewed by Teets et al) [13]. Thus, there is longstanding interest in the mechanisms that insects use to withstand cold temperatures in winter [6].

The RCH response has been evaluated in many insect species [13], including, *Liriomyza trifolii* (Burgess) and *L sativae* (Blanchard) (Diptera: Agromyzidae) which are economically important leafminer flies [14]. Both species are highly polyphagous and similar in morphology [15], life cycle and host range [16, 17]. *L*. *trifolii* and *L*. *sativae* are native to the Americas but have quickly spread to other regions of the world [18]. *L*. *trifolii* invaded mainland China in 2005 after *L*. *sativae* (1993), but it has displaced *L*. *sativae* and is now the dominant species in southern China [19, 20]. These species have complex interspecific interactions, and the displacement of one species by other has been observed in some regions [21–23]. It is thought that the displacement of *L*. *sativae* by *L*. *trifolii* in California is due to the high resistance of *L*. *trifolii* to pesticides [22, 24], while the displacement of *L*. *trifolii* by *L*. *sativae* in Japan has been hypothesized to be due to higher fecundity of *L*. *sativae* [25]. Displacements could also be caused by other environmental conditions such as temperature, which appears to be one of the major environmental factors affecting the distribution of *Liriomyza* spp. [26]. Variability in the expression of genes encoding heat shock proteins (*hsp)* in *L*. *huidobrensis* and *L*. *sativae* at various temperatures in laboratory conditions reflects differences in temperature tolerance, which may influence their natural geographical distribution [27, 28].

A number of studies have been conducted to characterize the mechanisms underlying RCH. For example, 37 differentially expressed genes (DEGs) were reported in *Drosophila melanogaster* in a 30-min recovery period after hardening [29]. However, transcriptional changes with RCH have also been reported in some studies without recovery period after cold hardening. Significant upregulation of transcripts encoding calcium/calmodulin protein kinase II was reported in the oriental fruit fly (*Bactrocera dorsalis*) after RCH treatment at 9 °C for 1.5 h [30]. The mRNA expression of heat shock protiens (*hsps*) in the rice water weevil (*Lissorhoptrus oryzophilus*) were found to be up-regulated after 2h exposure to 0 °C [31]. Pre-exposure to 0°C for 2 h significantly increased the expression levels of *hsps* in the western flower thrips (*Frankliniella occidentalis*) under cold shock treatment (−8°C for 2 h) [32]. Furthermore, RCH also involved in the upregulation of transcripts encoding metabolic enzymes, including glycerol and trehalose synthesis, in *Plutella xylostella* (after 7h exposure to 4 °C) and *Maruca vitrata* (after 1h exposure to mild low temperature) [12, 33]. However, in some studies when gene expression was measure immediately after hardening, no or very few DEGs were reported. In the absence of a recovery period, *D*. *melanogaster hsps* and *frost* were not differentially expressed during RCH treatment [34]. Similarly, among total of 219 genes, none were differentially regulated during RCH treatment at 0 °C for 1h in *Drosophila virilis*, whereas one (P5cr) upregulated and two downregulated genes (Eip 71CD and cwo) were identifies in *Drosophila montana* [35]. Consistent with a weak transcriptomic response to RCH in previous studies in *Drosophila* species, a study in the fly *Sarcophaga bullata* showed no changes in transcript abundance in response to 2h hardening under 0 °C [36]. In the above-mentioned studies, microarrays or selected genes were compared for expression; however, we are not aware of any previous reports where next generation sequencing (NGS) technology has been used to study RCH.

NGS technology has revolutionized the fields of genomics and transcriptomics and provides an opportunity to generate large-scale data rapidly and economically [37]. These technologies have been successfully used to detect and classify DEGs under various experimental conditions in many invasive species, including *Bemisia tabaci* [38], *Pomacea canaliculata* [39], *Bactrocera dorsalis* [40] and *Liriomyza spp*. [41, 42]. Transcriptomics is used to analyze systematic changes in gene expression and has been used to evaluate the response of insects to thermal stress [43, 44]. Large-scale transcriptional profiling in response to thermal stress has been undertaken for *Liriomyza* spp [42]; however, the mechanistic basis for RCH in *Liriomyza* species and how it contributes to interspecific differences has not been previously studied. We evaluated the RCH ability of *L*. *trifolii* and *L*. *sativae* and found that RCH is stronger and more durable in *L*. *sativae* pupae than pupae of *L*. *trifolii*. The survival rate of *L*. *sativae* under 2h cold shock was increased from 20% to 91% after pretreated under 1 °C for 4h, while in *L*. *trifolii*, the survival rate was increased from 20% to 60% after the same treatment. Also, the effect of RCH lasts for 4 h in *L*. *sativae* but only 2 h in *L*. *trifolii*. What’s more, supercooling point (SCP) of *L*. *sativae* was 2 °C more lowered than in *L*. *trifolii* after 4h cold hardening [14]. Based on these prior results, 7-d-old pupae that exhibited and showed obvious RCH effect in both *L*. *trifolii* and *L*. *sativae* were selected as material for this research.

In the present research, RNA-seq was used to identify transcriptional changes in both the species, that might explain differences in RCH in *L*. *trifolii* and *L*. *sativae*. Transcriptome data were validated by RT-qPCR. The results obtained in this study will further advance our understanding of RCH in these two closely-related species.

## Materials and methods

### Insects

Leaves infested with leafminers were collected from greenhouses in Yangzhou, China (32.39°N, 119.42°E) in 2019. As two closely related species with the same rates of larval-pupal-pharate adult development [45, 46], adults were identified as *L*. *trifolii* or *L*. *sativae* based on morphological characters. Both species were reared on kidney beans in growth chambers at 26°C with 16: 8 h light: dark photoperiod. Pupae were collected from leaves immediately after emergence and placed in plastic containers covered with gauze. They were maintained in growth chambers with their pupation time indicated. Seven-day-old pupae of both species, which were likely true pupae [47], were used for further experiments.

### Temperature treatments

Temperatures were selected based on our previous study [14]. Pupae of both species were exposed to the following conditions: control (maintained at 26°C), RCH (exposed to 1°C for 4 h), CS (cold shock, exposed to discriminating temperature for 2h, discriminating temperature; temperature caused 80 percent mortality, for *L*. *trifolii* -10.6, *L*. *sativae* -8.4°C), RCHCS (Acclimated for 4h at 1°C prior to exposure to discriminating temperature or cold shock). Each experiment contained three replicates per temperature (30 individuals/replication). Samples were frozen in liquid nitrogen immediately after treatment and were stored at -70°C until needed for RNA extraction.

### RNA extraction and transcriptome sequencing

Total RNA was isolated from pooled samples (*n* = 30) of *L*. *sativae*, and *L*. *trifolii* pupae, using the SV Total RNA isolation system (Promega, Fitchburg, WI, USA). The purity and integrity of RNA was determined as previously stated [48], and samples were chosen with *A*_260/280_ ratios of 1.8–2.2 and RNA integrity (RIN) values greater than 7.0. Three replications were used for each temperature treatment.

Transcriptome sequencing was provided by Biomarker Technologies Inc. (Beijing, China). Total RNA amount (1 μg per sample) was used as input material, and sequencing libraries were generated using NEBNext^®^Ultra^™^ RNA Library Prep Kit for Illumina^®^ (NEB, USA). The clustering of index-coded samples was performed on a cBot Cluster Generation System using TruSeq PE Cluster Kit v3-cBot-HS (Illumina) according to the manufacturer’s instructions. After cluster generation, the library preparations were sequenced on an Illumina Hiseq 2000 platform and paired-end reads were generated. RNA-seq data were uploaded to the National Center for Biotechnology Information (NCBI) as Sequence Read Archive no. PRJNA756384.

### Quality control and transcriptome assembly

Prior to transcript assembly, clean data were obtained by removing adapter sequences, artificially introduced bases and low-quality reads using Trimmomatic 0.32 (http://www.usadellab.org/cms/index.php?page=trimmomatic). Q20 and Q30 scores, GC-content and sequence duplications in the clean data were calculated, and all analyses were based on clean, high quality data. Transcriptome assembly was accomplished using Trinity [49], with min_kmer_cov set to 2 and all other parameters set as default. The unigene library of the two species were first assembled to obtain individual unigene databases; the general unigene library was obtained by clustering these two individual databases of *L*. *sativae*, and *L*. *trifolii* through CD-Hit to facilitate comparison of expression patterns [50]. Principle component analysis (PCA) was achieved by using the online platform at www.biocloud.net (Biomarker Technologies Inc., Beijing, China).

### Functional annotation of genes

Gene function was annotated based on the following databases: NR (NCBI nonredundant protein sequences); Pfam (Protein family); COG (Clusters of Orthologous Groups of proteins); Swiss-Prot (manually annotated and reviewed protein sequence database); KEGG (Kyoto Encyclopedia of Genes and Genomes) and GO (Gene Ontology). Software including BLAST, KOBAS2. HMMER were used for annotation.

### Differential gene expression analysis

Read counts were mapped and then fragments per kilobase of transcript sequence per million (FPKM) nucleotides were calculated. The assembled transcriptomes of treated samples were compared with each other, and differential expression was detected using the DESeq2 R package as described by Varet et al [51]. The resulting P values were adjusted using the Benjamini-Hochberg procedure for controlling false discovery rates (FDR). An FDR<0.05 and fold-change |FC| ≥ 2 were used to evaluate significant differences in gene expression. Gene Ontology (GO) enrichment analysis of the differentially expressed genes (DEGs) was implemented by Kolmogorov–Smirnov test based on the topGO R package (https://bioconductor.org/packages/release/bioc/html/topGO.html). KEGG enrichment analysis of DEGs was carried out with KOBAS software. The corrected *P*-value were calculated with hypergeometric test as a service provided by Biomarker technologies Inc (Beijing, China).

### Real-time quantitative PCR

Based on sequencing results, eight unigenes were chosen and used to validate RNA seq data by real-time quantitative PCR (RT-qPCR). Primers were designed by Primer Premier v. 5.0 (http://www.premierbiosoft.com/primerdesign/). cDNA was synthesized in 20-μl reaction volumes as described previously and *Actin* was used as a reference gene [43]. Relative changes in gene expression were analyzed using the 2^−ΔΔCt^ method [52]. Each PCR reaction included three replicates. Data were analyzed with one-way ANOVA, and Tukey’s multiple comparison test using SPSS v. 16.0 (SPSS, USA). Differences were considered statistically significant when *P* < 0.05.

## Results

### Overview of RNA sequencing data

A total of 215.08 Gb of clean reads was obtained with Q30 values ≥ 92.30% and GC content ranging from 38.59–39.70% (Table 1). In total, 93,166 unigenes were identified with an average length of 960 bp and N50 length of 2,075 bp. For functional classification, 10,740, 17,801, 14,935, 21,649, 23,747, 18,427, 31,539 and 29,404 unigenes were mapped to COG, GO, KEGG, KOG, Pfam, Swiss-Prot, eggNOG and NR databases respectively (Table 2).

**Table 1:** Features of *L*. *sativae* and *L*. *trifolii* in response to RCH.

Species	Samples	Clean read number	Clean base number	GC content (%)	% ≥ Q30
*L*. *sativae*	Ck1	31,089,426	9,294,158,712	39.39	93.07
	Ck2	28,830,373	8,629,916,894	39.26	92.72
	Ck3	26,978,296	8,069,176,154	39.51	92.30
	RCH1	28,947,942	8,659,897,872	39.59	93.25
	RCH2	26,895,638	8,046,967,212	39.54	92.76
	RCH3	29,754,079	8,896,382,678	39.63	92.96
	CS1	31,742,685	9,496,153,320	39.55	92.65
	CS2	28,361,877	8,498,733,786	39.69	92.89
	CS3	39,003,142	11,664,771,094	39.57	92.69
	RCH-CS1	29,780,455	8,903,886,606	39.61	92.44
	RCH-CS2	28,294,918	8,467,552,974	39.50	92.31
	RCH-CS3	27,572,446	8,259,527,012	39.57	92.71
*L*. *trifolii*	Ck1	28,493,041	8,531,547,414	39.58	93.16
	Ck2	34,398,959	10,289,475,664	39.79	93.19
	Ck3	33,024,729	9,884,874,734	39.47	93.37
	RCH1	30,708,409	9,193,798,506	39.62	94.28
	RCH2	29,590,692	8,854,262,332	38.59	93.47
	RCH3	29,130,331	8,715,930,284	39.60	93.91
	CS1	28,238,996	8,442,743,612	39.50	93.74
	CS2	28,156,922	8,415,800,272	39.41	93.91
	CS3	30,001,073	8,967,111,816	39.67	93.94
	RCH-CS1	29,322,131	8,767,003,242	39.45	93.96
	RCH-CS2	29,173,223	8,711,675,602	39.70	93.70
	RCH-CS3	31,468,767	9,417,706,950	39.28	94.57

CK, control (25 °C); RCH, Rapid Cold Hardening (1°C for 4h); CS, Cold shock (temp gives 80% mortality) and RCHCS (1°C for 4h+ 2h exposure to CS).

**Table 2:** Summary of statistics and annotation.

Sequencing/annotation	Data summary
Total number of unigenes	93,166
Mean length of unigenes (bp)	960.32
N50 length of unigenes (bp)	2,075
COG annotated	10,740
GO annotated	17,801
KEGG annotated	14,935
KOG annotated	21,649
Pfam annotated	23,747
Swiss-Prot annotated	18,427
eggNOG annotated	31,539
NR annotated	29,404
All annotated	34,303

### Analysis of differentially expressed genes (DEGs)

Gene expression changes were analyzed by comparing leafminers kept at room temperature to leafminers acclimated at 1°C for 4h (LtCK vs. LtRCH and LsCK vs. LsRCH). Gene expression was also compared for leafminers exposed to a 2 h cold shock (CS) to leafminers acclimated at 1°C for 4 h prior to the 2 h cold shock (LtCS vs. LtRCHCS and LsCS vs. LsRCHCS).

### Differential gene expression between controls and RCH

**Fig 1:**
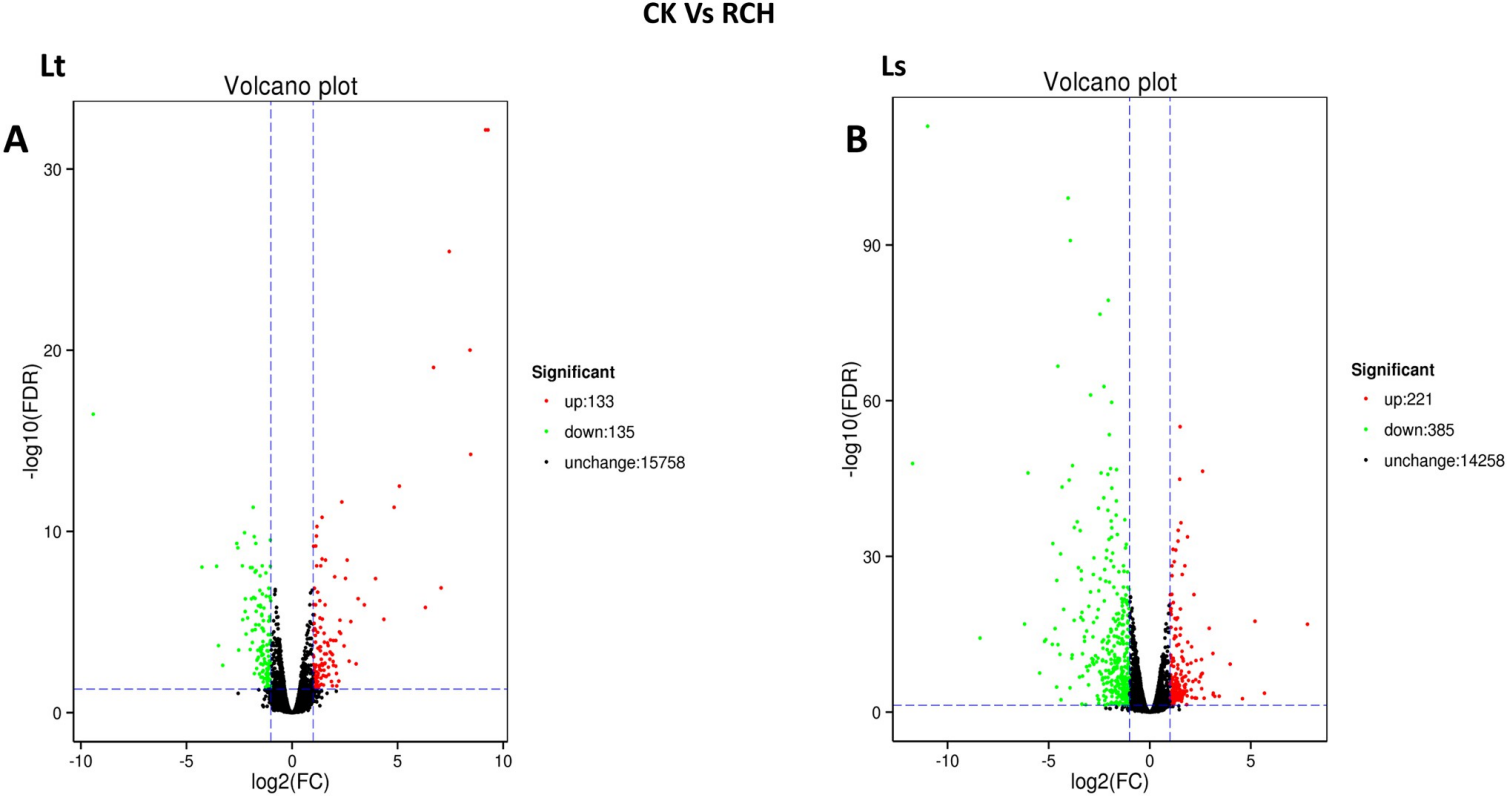
Differentially expressed genes (DEGs) in *L*. *trifolii* and *L*. *sativae* exposed to thermal stress. (A) Volcano plot of *L*. *trifolii* of RCH vs. control pupae. (B) Volcano plot of *L*. *sativae* of RCH vs. control pupae. The y-axis represents -log10 significance, whereas the x-axis represents log2-fold change. Red dots indicate induced unigenes, whereas green dots indicate repressed unigenes. All values were obtained based on discriminative power (|fold change| ≥ 2.0 and FDR ≤ 0.05).

Pupae of both *L*. *trifolii* and *L*. *sativae* showed transcriptional regulation after being transferred to 1 °C for 4-h. The number of the differentially expressed genes in pupae of *L*. *sativae* is much more than that in *L*. *trifolii*. A total of 268 and 606 unigenes were differentially expressed in *L*. *trifolii* and *L*. *sativae*, respectively (Fig 1). There were 135 and 133 unigenes down and up-regulated unigenes, respectively, in *L*. *trifolii* after a 4 h acclimation period (Fig 1A). In *L*. *sativae*, 221 unigenes were up-regulated and 385 were down-regulated (Fig 1B).

To better understand gene function, DEGs were further annotated using COG and KEGG databases. In both species, carbohydrate transport and metabolism were one of the most significantly enriched COG terms. (S2 Fig). The top 20 enriched KEGG pathways after acclimation in *L*. *trifolii* and *L*. *sativae* are shown in S3 Fig. Among them, the most significantly enriched was “galactose metabolism” in *L*. *trifolii* (corrected *P* value = 0.0001), whereas the most enriched pathway in *L*. *sativae* pupae was “peroxisome” (corrected *P*-value = 0.0001). The top 10 upregulated DEGs in *L*. *trifolii* after RCH are shown in S1 Table. The most upregulated gene was annotated as ‘cytochrome c oxidase subunit’, which is related to energy production and conversion. Other than the top10, genes related to many other metabolic pathways are involved as well, such as fatty acid synthase, glucose dehydrogenase, cuticular protein and so on. The top 10 upregulated genes in *L*. *sativae*, included keratin, type II cytoskeletal 1b, and bumetanide-sensitive sodium-(potassium)-chloride cotransporter, which is involved in inorganic ion transport and metabolism. Besides, eleven DEGs were annotated as cuticular protein unigenes (S1 Table).

### Differential genes expression between CS and RCHCS

**Fig 2:**
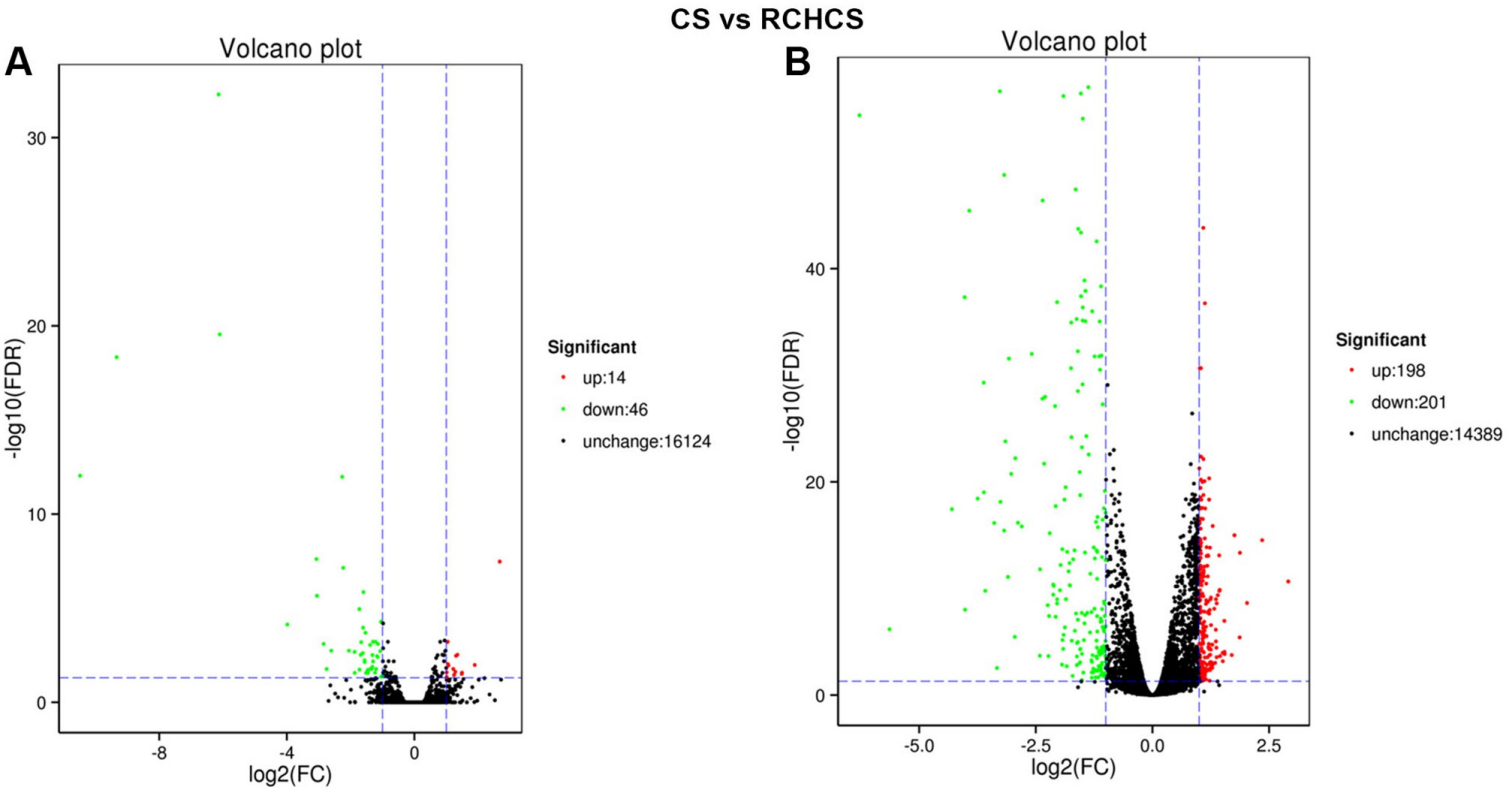
Differentially expressed genes (DEGs) in *L*. *trifolii* and *L*. *sativae* exposed to thermal stress. (A) Volcano plot of *L*. *trifolii* of CS vs. RCHCS pupae. (B) Volcano plot of *L*. *sativae* of CS vs. RCHCS pupae. The y-axis represents -log10 significance, whereas the x-axis represents log2-fold change. Red dots indicate induced unigenes, whereas green dots indicate repressed unigenes. All values were obtained based on discriminative power (|fold change| ≥ 2.0 and FDR ≤ 0.05).

Same with the previous comparison, in this comparison of CS and RCHCS, pupae of *L*. *sativae* exhibited more DEGs than pupae of *L*. *trifolii* (Fig 2). Only 60 unigenes were found to be differentially expressed in *L*. *trifolii*; 46 were down- and 14 were up-regulated, respectively (Fig 2A). In contrast, 399 DEGs were identified in *L*. *sativae*, and 198 were up-regulated and 201 were down-regulated (Fig 2B). The most significantly enriched COG term in both species was “post-translational modification, protein turnover, chaperones” (S4 Fig). In *L*. *trifolii*, only three KEGG pathways were identified and the most significantly enriched pathway was “biosynthesis of unsaturated fatty acids” (corrected *P* value = 0.03). In *L*. *sativae*, 41 pathways were identified and the most enriched pathway was “glutathione” (corrected *P* value = 0.008) (S5 Fig). In *L*. *trifolii* pupae treated by RCHCS, only seven genes were significantly up-regulated when compared to a cold-shocked (CS) pupae without hardening. Among these, the most upregulated gene is related to cytochrome P450, which showed a 1.28-fold higher expression level. In *L*. *sativae*, the most highly upregulated genes included apolipoprotein, thioredoxin protein and serine protease easter, which are involved in several different pathways. Furthermore, a number of genes related to cuticular proteins were differently expressed when comparing CS with RCHCS. The most highly up-regulated genes in the comparison of RCHCS and CS in the two Liriomyza spp. are shown in S2 Table.

### Classification of common DEGs in *L*. *trifolii* and *L*. *sativae*

**Fig 3:**
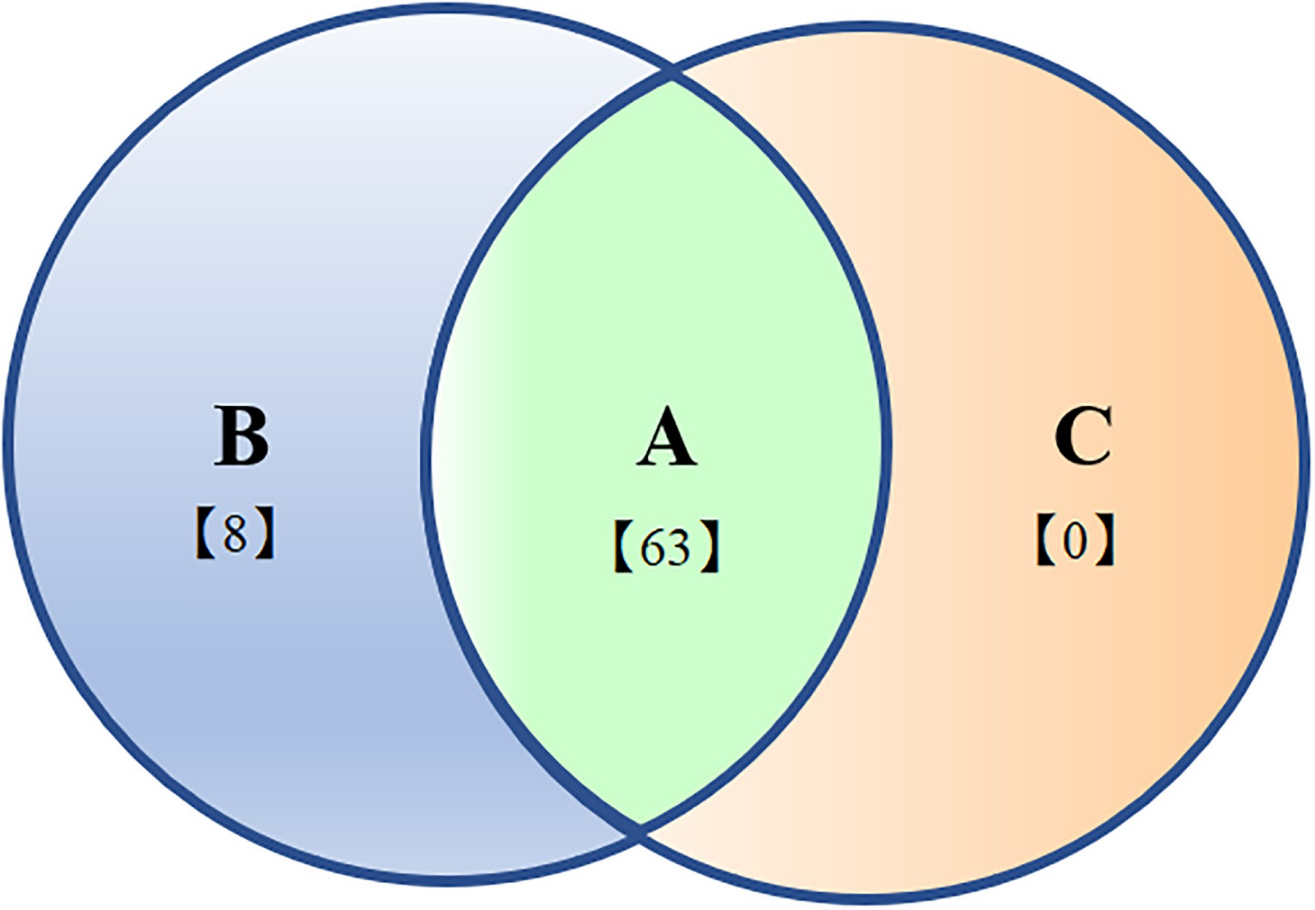
Classification of Co-DEGs in leaminers based on their expression patterns. The expression patterns of Co-DEGs are classified into three categories: A, Co-DEGs showed same expression pattern in both species; B, Co-DEGs up-regulated in *Liriomyza trifolii* but down-regulated in *Liriomyza sativae*; C, Co-DEGs down-regulated in *Liriomyza trifolii* but up- regulated in *Liriomyza sativae*.

Based on gene expression changes in response to different RCH treatments, the DEGs common (Co-DEGs) to both *Liriomyza* spp. were classified into three types. Type 1 included Co-DEGs with similar expression pattern in both species, type 2 consisted of Co-DEGs up-regulated in *L*. *trifolii* but down-regulated in *L*. *sativae*, and type 3 included Co-DEGs down-regulated in *L*. *trifolii* but up-regulated in *L*. *sativae* (Fig 3).

A total of 71 genes were differentially expressed among the two leafminer species after a 4-h cold hardening period, and a heatmap is shown in S6 Fig. No genes were identified showing up-regulation in *L*. *sativae* but down regulation in *L*. *trifolii*. Sixty-three Co-DEGs were categorized as Type I and the top five upregulated genes in the two species are listed (S3 Table). These genes are related to several different clusters, including ‘secondary metabolite biosynthesis, transport and catabolism’, ‘Posttranslational modification, protein turnover, chaperones’ and ‘Carbohydrate transport and metabolism’. The top five genes down-regulated in both species are related to cytochrome P450, cuticle, histidine-rich glycoprotein, glucose dehydrogenase, mastermind-like protein and elongation of very long chain fatty acids protein. Eight genes showed opposite expression patterns in *L*. *sativae* and *L*. *trifolii*, including genes associated with chymotrypsin, defensin-A and cell wall-associated hydrolase.

When comparing RCHCS and CS, only 17 co-DEGs were identified in both species (S6 Fig) and these genes had identical expression patterns in the two species. Among these, four genes were up-regulated; one DEG assigned to solute carrier family 22 and another was a histidine-rich protein. Thirteen DEGs were down regulated in both *L*. *sativae* and *L*. *trifolii*, and these included DEGs assigned to cationic amino acid transporter, cytochrome P450, delta (9)-fatty-acid desaturase and glucose dehydrogenase which are related to lipid transport and metabolism.

### Data validation by RT-qPCR

**Fig 4:**
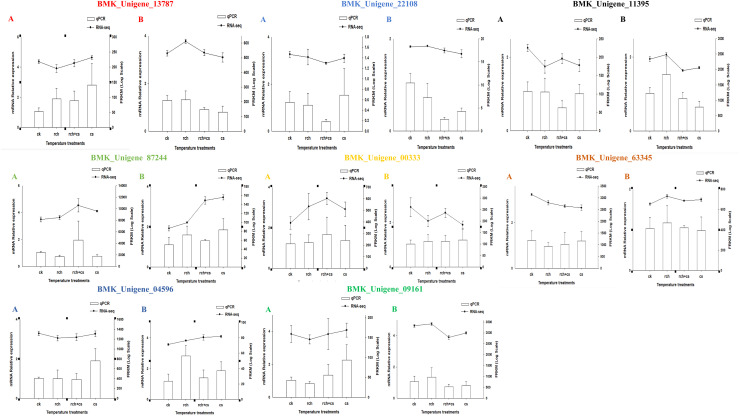
Validation of gene expression patterns by RT-qPCR. Means (±SE) were used to determine transcript levels with the 2−ΔΔCt method. One-way analysis of variance (ANOVA) was used to analyze the relative expression levels of DEGs. For the ANOVA, data were tested for homogeneity of variances and normality. Tukey’s multiple range test was used for pairwise comparison for mean separation (P < 0.05). The y-axes indicate log-transformed FPKM values (right) and log-transformed relative expression (left).

To ensure the reliability of RNA-seq data, RT-qPCR was used to validate eight DEGs with distinct expression patterns including three cuticular protein genes (87244, 63345, 09161), two *hsp* genes (13787, 00333), a gene encoding anti-freeze protein (04596), a gene encoding glyceraldehyde-3-phosphate dehydrogenase (11395), and one gene involved in ATP synthesis (22108). The expression levels obtained by RT-qPCR correlated with those observed by RNA-Seq (*L*. *trifolii*: R^2^ = 0.72, *p* < 0.05, *L*. *sativae*: R^2^ = 0.63, *p* < 0.05), thus confirming the reliability of the RNA-Seq data (Fig 4, S1 Fig), indicating that transcriptome data are reliable.

## Discussion

A longer or shorter exposure to mild temperature can potentially trigger acclimation responses of insects [53, 54]. 7-d-old pupae exhibited and showed obvious RCH effect in both *L*. *trifolii* and *L*. *sativae* based on our previous research were selected as material for this study [14]. Due to global warming, fluctuations in daily temperature have increased [55], resulting in enhanced temperature variation in winter hibernacula, and more freeze-thaw events [56]. Abnormal temperature fluctuations and, increased thermal instability in early spring and late autumn has made, RCH an important factor in insect survival [57]. As pests that usually overwinter in the pupal stage [58], RCH ability will be contribute to the overwinter survival of *L*. *trifolii* and *L*. *sativae*. In this study, we used NGS technology to investigate the pathways involved in RCH and discover differences in the molecular response of *L*. *trifolii* and *L*. *sativae* to RCH. When comparing RCH vs. control group and CS vs. RCHCS, more differentially expressed genes were observed in *L*. *sativae* than in *L*. *trifolii*. This was especially true in the CS vs. RCHCS comparison, where nearly 400 DEGs were identified in *L*. *sativae*. In contrast only 60 DEGs were identified in *L*. *trifolii*. These results indicate that *L*. *sativae* is more responsive to RCH than *L*. *trifolii* at the molecular level and may explain why pupae of *L*. *sativae* have a stronger response to rapid cold hardening than pupae of *L*. *trifolii* [14].

### Species differences in the molecular mechanism of RCH

In this study, RCH resulted in transcriptional changes in both *L*. *trifolii* and *L*. *sativae*. Studies on *Frankliniella occidentalis* and *B*. *dorsalis* also reported transcriptional changes associated with RCH including the upregulation of *hsp* and transcripts encoding calcium/calmodulin protein kinase II [30, 32]. Transcripts encoding metabolic enzymes such as, glycerol and trehalose synthesis were elicited by RCH and have been reported in some lepidopteran pests [12, 33]. However, there were also some previous studies on the molecular mechanisms of RCH reported that RCH do not permit transcriptional activity [34–36]. Despite the obvious effects of RCH on survival, it elicited few changes in the abundance of detectable proteins in *Drosophila* [59]. Our research, in combination with other studies, indicates that the mechanism of RCH varies greatly in different species.

The genes exhibiting the most dramatic changes in expression level after cold hardening were different in *L*. *trifolii* and *L*. *sativae*. For example, a gene associated with trypsin was, highly upregulated in *L*. *trifolii* after a 4 h period of hardening but showed decreased tendency of expression level in *L*. *sativae*. Differences in gene expression in response to the same thermal treatments have been noted in other related species, including corals, fish, planthoppers and fruit flies [60–63].

Differences between the two *Liriomyza* species were also evident in our KEGG analysis. The most enriched pathway in *L*. *trifolii* after a 4 h acclimation was galactose metabolism. Galactose can be converted to glucose-6-phosphate in most organisms when glucose, an important cryoprotectant for insects, is not available [64, 65]. The switch to the galactose pathway is highly related to glucose levels [66, 67]; thus, the enrichment of galactose metabolism may indicate that *L*. *trifolii* pupae are utilizing galactose instead of glucose during RCH, which warrants further study. The most enriched pathway in *L*. *sativae* was ‘peroxisome’, which is an organelle involved in the regulation of oxidative stress and the catabolism of long chain fatty acids [68]; the latter is significant because shorter-chain fatty acids are presumably more important in membrane fluidity than longer chain fatty acids [69, 70]. Prior research has shown that organisms adjust the membrane composition to maintain fluidity at different temperatures, which is an important component of cold hardiness [71, 72]. Changes in the average chain length of fatty acids as a component of RCH warrants further investigation.

### Similarities in the molecular basis of RCH in *Liriomyza* species

Despite differences in *L*. *trifolii* and *L*. *sativae*, DEGs common to both were identified. There were DEGs which were regulated commonly in *L*. *trifolii* and *L*. *sativae* during temperature treatments. Total of 71 and 17 shared DEGs were identified in CK vs. RCH and CS vs. RCHCS comparisons, respectively, and these DEGs related to P450, cuticular protein and fatty acids. These common DEGs were regulated similarly in *L*. *trifolii* and *L*. *sativae* during temperature treatments; this may reflect global changes in gene expression in response to RCH. While the other common DEGs which showed the contrast expression patterns in different species stand for the interspecific divergence of the transcriptional mechanism of low-temperature adaptation.

In both species, cuticular genes were modulated by exposure to a 4 h hardening period. Insect cuticles support body structure and provide protection from desiccation, insecticides, parasites and pathogens [73]. Cold-responsive cuticular genes have been reported in many insects including beetles [74, 75], carpenter moths [76], plant-hoppers [62], stick insects [77], locusts [78], wasps [79], and flies [29]. Increased expression of cuticular protein has been recorded in diapausing *D*. *melanogaster* [80], and the fortification of the cuticle by cuticular proteins was detected in overwintering *Cucujus clavipes puniceus* [74]. It is unclear how the changes in the cuticular proteins help insects withstand cold stress; for example, these proteins may help prevent ice nucleation by fortifying the cuticle of overwintering insects [74]. It is also possible that leafminers use RCH as a signal of imminent cold stress, and the regulation of cuticular genes during RCH may help the insect prepare for potential cold shock. Further work is required to identify whether the cuticular structure is modified by cold hardening, and how cuticular proteins contribute to cold tolerance.

Multiple P450 genes were expressed after a 4-h acclimation in both *Liriomyza* species. The cytochrome P450 family is involved in oxidative metabolism, and activity in this gene family has been associated with temperature stress [81]. The cytochrome P450 associated transcripts were enriched in *Laodelphax striatellus* (small brown planthopper) exposed to low temperatures, suggesting that the regulation of cytochrome P450 participated in enhanced cold tolerance of this species [62]. Upregulation of cytochrome P450s during temperature stress was also reported in fall armyworm, *Spodoptera frugiperda* [82]. Thermal stress may trigger the production of reactive oxygen species and lead to oxidative stress; consequently, the activity of antioxidant systems is boosted to prevent oxidative damage [62].

Genes related to carbohydrate transport and metabolism were also enriched in both *Liriomyza* spp. Carbohydrates are used as a source of energy and are transformed into energy reserves (i.e., glycogen). Previous studies have reported that carbohydrate synthesis and catabolism are critical for maintaining osmotic balance during stressful temperatures [83]. These processes are also responsible for providing energy during physiological and biochemical reactions inside cells [84]. In both *L*. *trifolii* and *L*. *sativae*, genes responsible for temperature stress are regulated after a 4-h acclimation. Genes related to carbohydrate synthesis and catabolism were differentially expressed when comparing insects that been cold-hardened prior to cold stress with those directly exposed to extreme cold stress. These results suggest that the abundance of the transcripts involved in metabolic energy and post-translational modification are commonly related to the cold stress adaptation in *L*. *trifolii* and *L*. *sativae*.

## Conclusion

**Fig 5:**
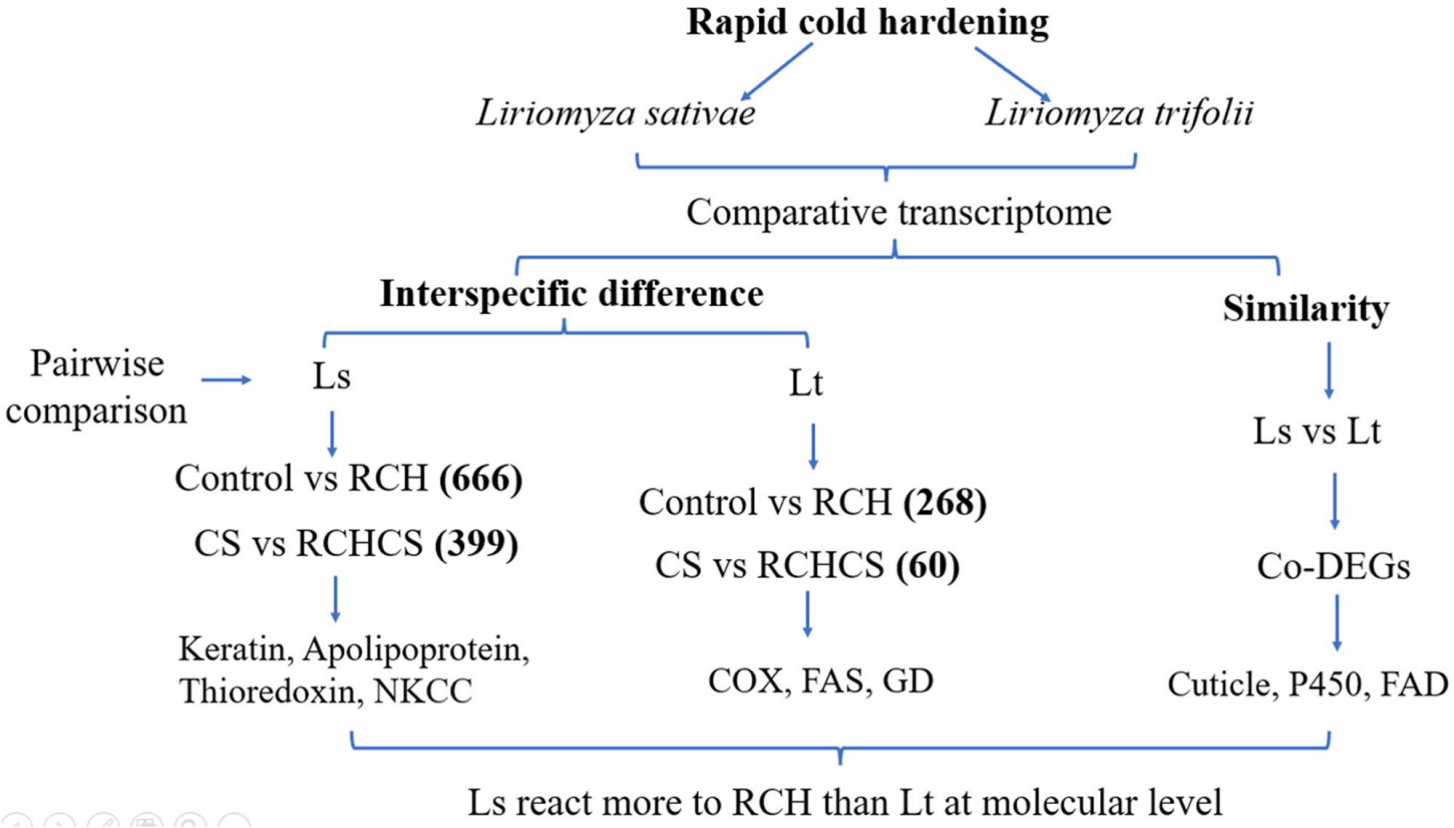
The potential molecular mechanisms for interspecific competition of two leafminer flies based on comparative transcriptome analysis.

Our transcriptomic data suggest that transcripts abundance of RCH varies in the pupae of *L*. *trifolii* and *L*. *sativae*, which helps explain the different magnitude of responses to RCH between *L*. *trifolii* and *L*. *sativae*. The two species also share common DEGs including genes related to cuticular protein, detoxification, and the transport and metabolism of carbohydrates and lipids; these may have a key role in the adaptation to low-temperatures (Fig 5). Although our research provides an invaluable dataset to understand the RCH phenomena at transcriptome level, the downstream processes of RCH that confer cold tolerance require further investigation.

## Supporting information

S1 Fig Linear correlation of analysis of RNA-seq and RT-qPCR data of 8 randomly selected genes. (A) *L*. *trifolii* and (B) *L*. *sativae*.
(PNG)

S2 Fig Functional classification of DEGs into clusters of orthologous groups (COG). (A) *L*. *trifolii* and (B) *L*. *sativae* of control vs RCH treatments.
(JPG)

S3 Fig The twenty most enriched KEGG pathways among DEGs in Control vs RCH (A) *L*. *trifolii* and (B) *L*. *sativae* pupae. The Rich factors represent the ratio of DEG numbers vs. the number of genes annotated in the pathway. Larger Rich factors indicate a greater level of enrichment. The q values are corrected P values ranging from 0 to 1, with lower values indicating greater enrichment.
(JPG)

S4 Fig Functional classification of DEGs into clusters of orthologous groups (COG). (A) *L*. *trifolii* and (B) *L*. *sativae* of CS vs RCHCS treatments.
(JPG)

S5 Fig The twenty most enriched KEGG pathways among DEGs in CS vs RCHCS (A) *L*. *trifolii* and (B) *L*. *sativae* pupae. The Rich factors represent the ratio of DEG numbers vs. the number of genes annotated in the pathway. Larger Rich factors indicate a greater level of enrichment. The q values are corrected P values ranging from 0 to 1, with lower values indicating greater enrichment.
(JPG)

S6 Fig Differential gene expression analysis among two leafminer species. (A) Venn plot of DEG numbers of CK vs RCH among the two species. (B) Heatmap of differentially expressed genes in Control vs RCH. (C) Venn plot of DEG numbers Cs vs RCHCS among the two species. (D) Heatmap of differentially expressed genes in CS vs RCHCS. Colour scale from red to green indicates log2 transcription ratios from 0 to 2. Abbreviations: Lsp, L. sativae pupae; Ltp, L. trifolii pupae; CK, control; RCH, rapid cold hardening; CS, cold shock.
(JPG)

S1 Table Upregulated (log2), annotated DEGs in control vs RCH of both species. (DOCX)

S2 Table Upregulated (log2), annotated DEGs in CS vs RCH of both species. (DOCX)

S3 Table Common upregulated (log2) and down regulation of annotated DEGs in Control vs RCH and CS vs RCHCS. (DOCX)
