# Supplementary figures and images for: Comparative transcriptome analysis of Liriomyza trifolii (Burgess) and Liriomyza sativae (Blanchard) (Diptera: Agromyzidae) in response to rapid cold hardening
Source: PLoS One. 2022 Dec 15;17(12):e0279254. doi: 10.1371/journal.pone.0279254 (PMC9754249; doi:10.1371/journal.pone.0279254)

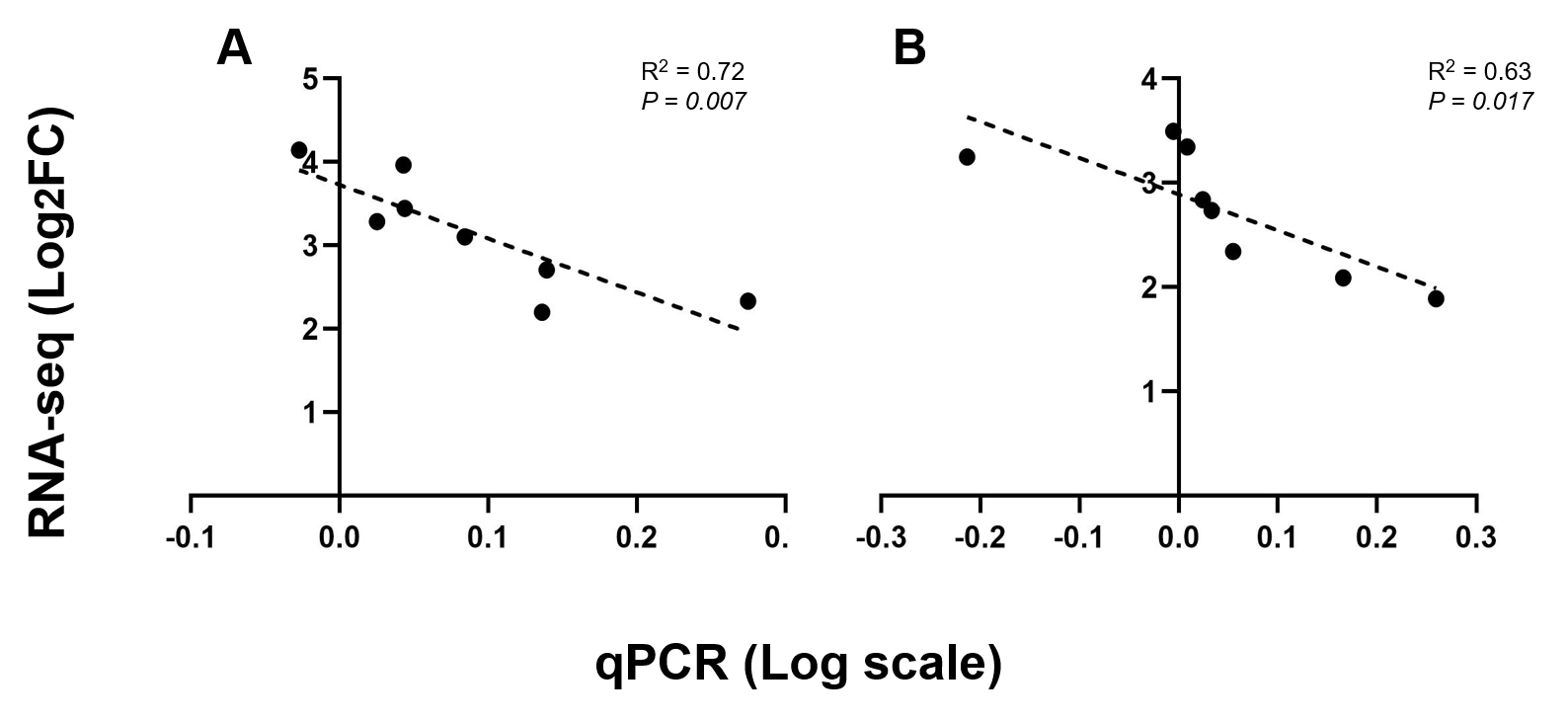

Supplement: S1 Fig — (A) L. trifolii and (B) L. sativae. (PNG) [file pone.0279254.s001.png]

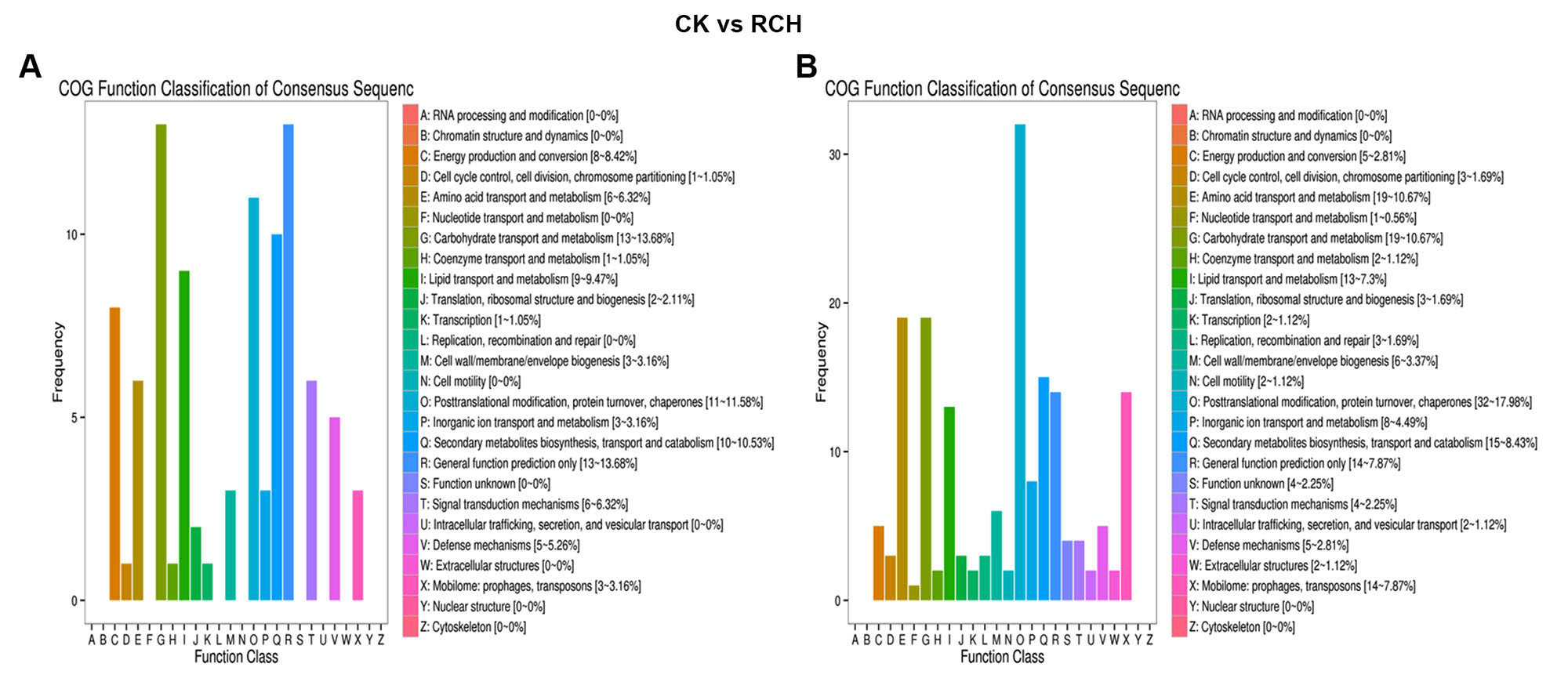

Supplement: S2 Fig — (A) L. trifolii and (B) L. sativae of control vs RCH treatments. (JPG) [file pone.0279254.s002.jpg]

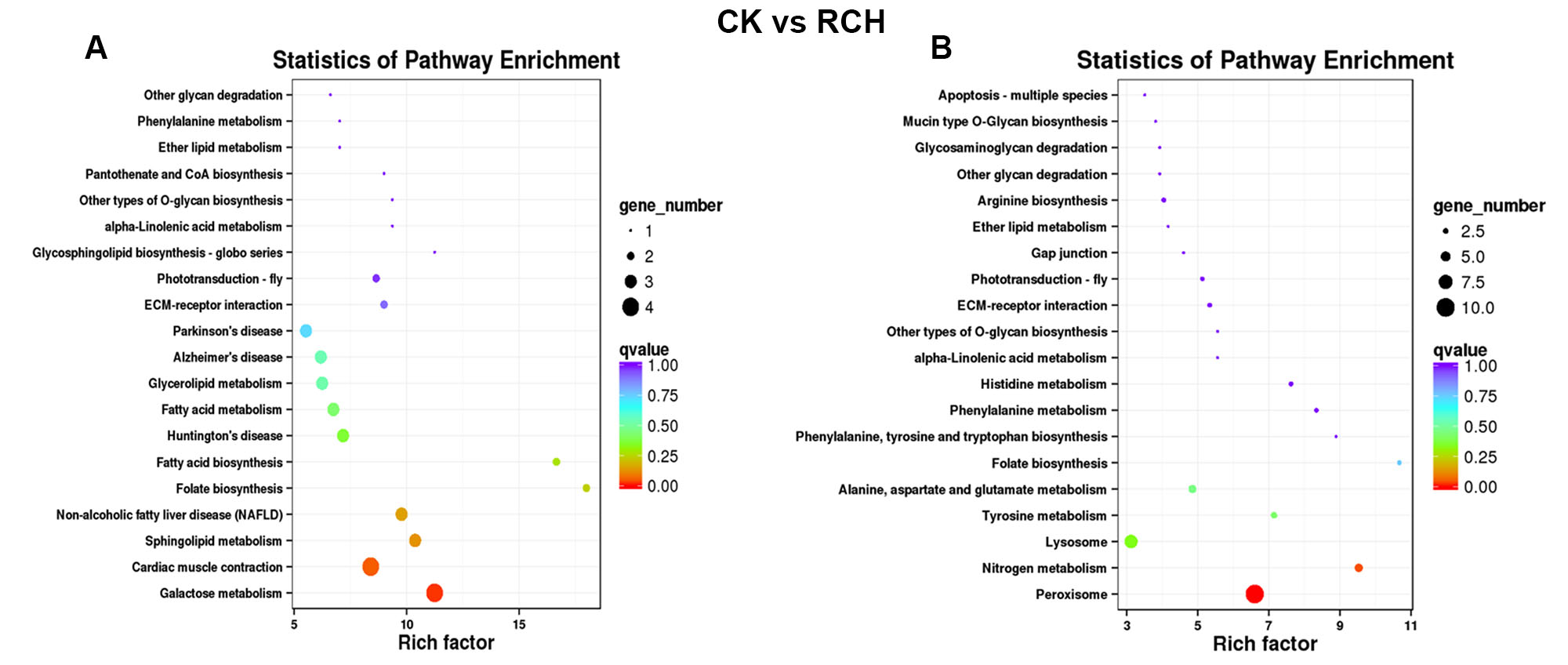

Supplement: S3 Fig — The Rich factors represent the ratio of DEG numbers vs. the number of genes annotated in the pathway. Larger Rich factors indicate a greater level of enrichment. The q values are corrected P values ranging from 0 to 1, with lower values indicating greater enrichment. (JPG) [file pone.0279254.s003.jpg]

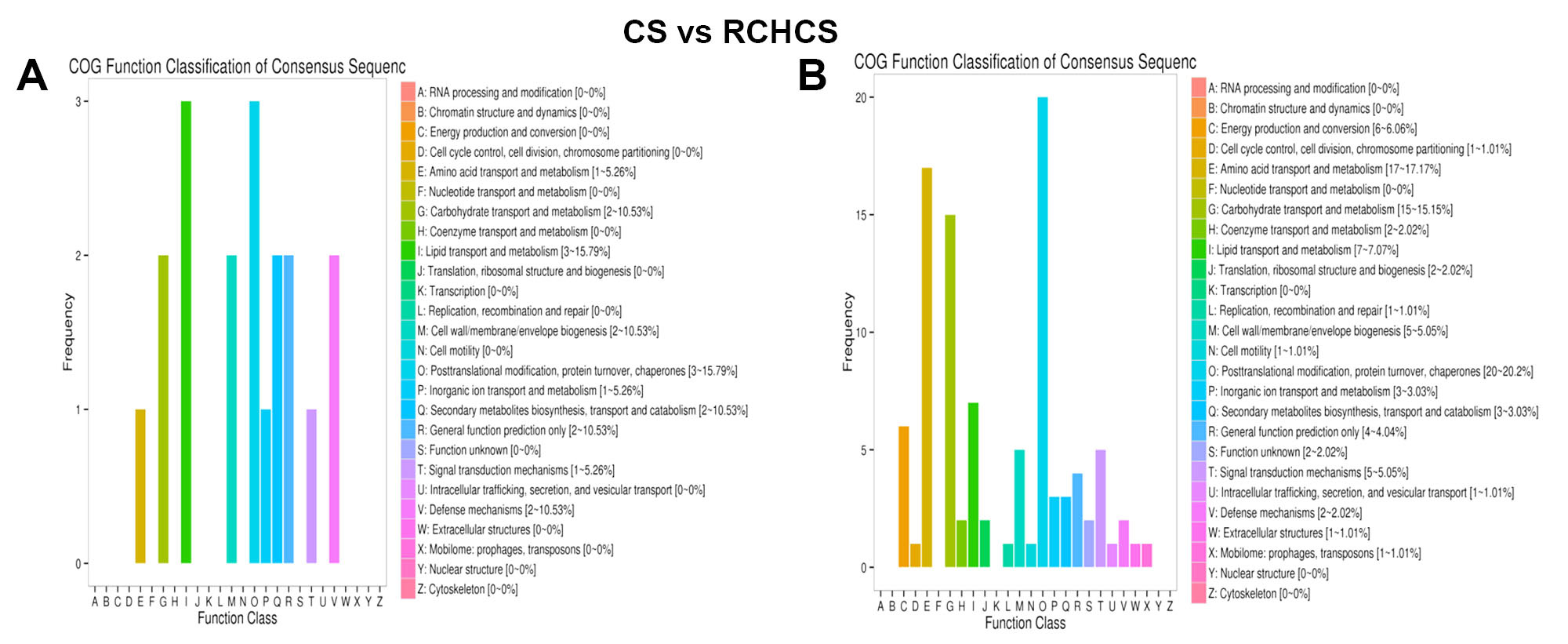

Supplement: S4 Fig — (A) L. trifolii and (B) L. sativae of CS vs RCHCS treatments. (JPG) [file pone.0279254.s004.jpg]

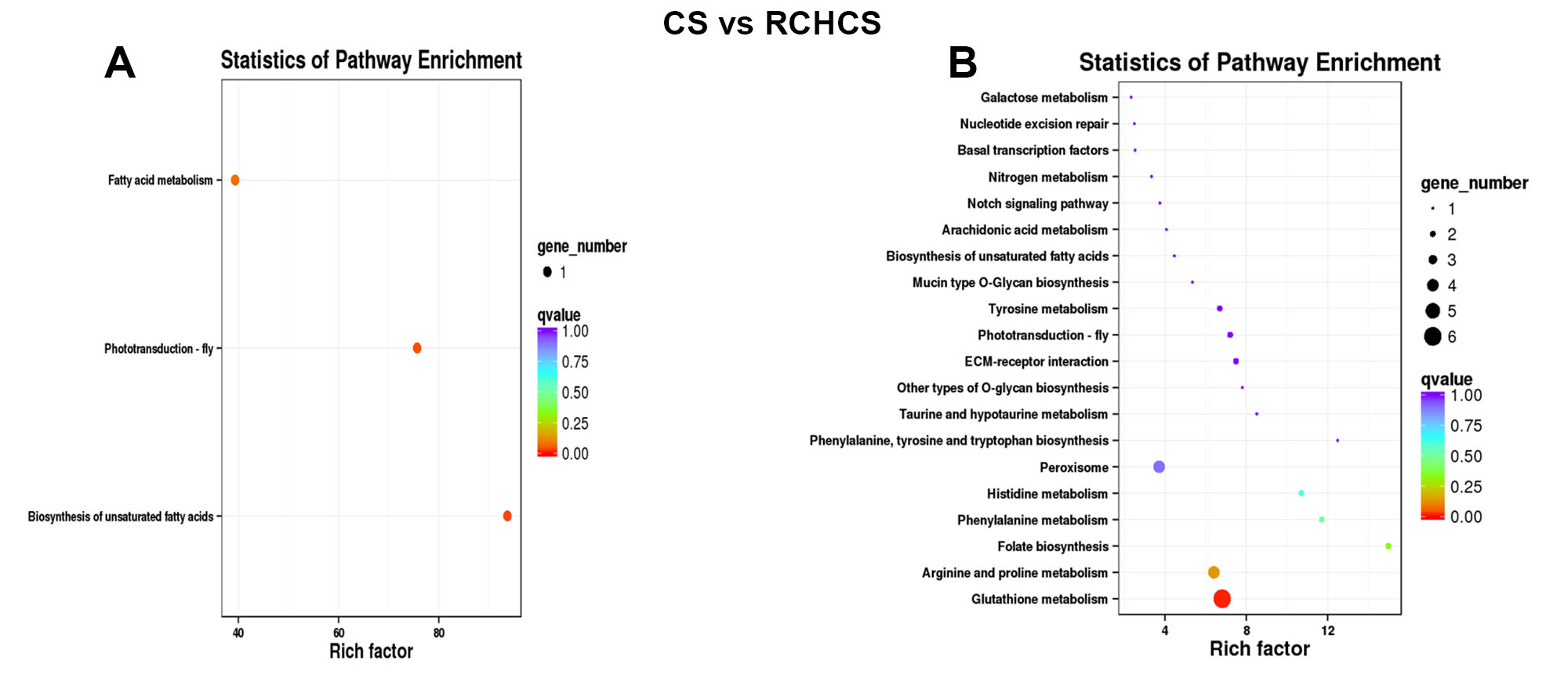

Supplement: S5 Fig — The Rich factors represent the ratio of DEG numbers vs. the number of genes annotated in the pathway. Larger Rich factors indicate a greater level of enrichment. The q values are corrected P values ranging from 0 to 1, with lower values indicating greater enrichment. (JPG) [file pone.0279254.s005.jpg]

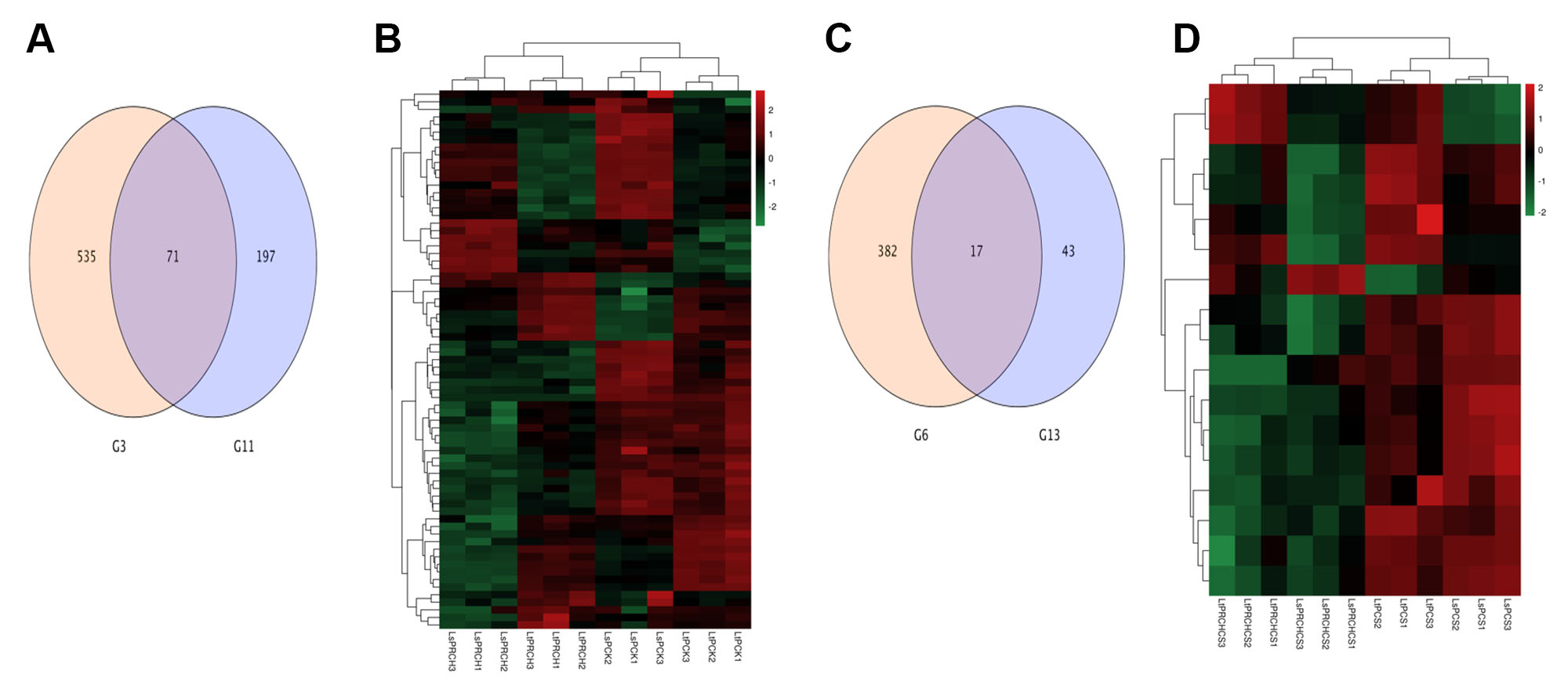

Supplement: S6 Fig — (A) Venn plot of DEG numbers of CK vs RCH among the two species. (B) Heatmap of differentially expressed genes in Control vs RCH. (C) Venn plot of DEG numbers Cs vs RCHCS among the two species. (D) Heatmap of differentially expressed genes in CS vs RCHCS. Colour scale from red to green indicates log2 transcription ratios from 0 to 2. Abbreviations: Lsp, L. sativae pupae; Ltp, L. trifolii pupae; CK, control; RCH, rapid cold hardening; CS, cold shock. (JPG) [file pone.0279254.s006.jpg]
