# Supplementary material for: Comparative transcriptome analysis of Liriomyza trifolii (Burgess) and Liriomyza sativae (Blanchard) (Diptera: Agromyzidae) in response to rapid cold hardening
Source: PLoS One. 2022 Dec 15;17(12):e0279254. doi: 10.1371/journal.pone.0279254 (PMC9754249; doi:10.1371/journal.pone.0279254)
Supplement: S1 Table — (DOCX) [file pone.0279254.s007.docx]

**S1 Table**. Upregulated (log2), annotated DEGs in control vs RCH of both species

| #ID | FDR | Log_2_FC | NR Annotation | |
| --- | --- | --- | --- | --- |
| Lt |  |  |  | |
| BMK_Unigene_65116 | 6.93E-33 | 9.164331066 | hypothetical protein NEMVEDRAFT_v1g156779 [Nematostella vectensis] |  |
| BMK_Unigene_92261 | 5.61E-15 | 8.456116537 | cytochrome c oxidase subunit I, partial (mitochondrion) [Gasterophilus pecorum] |  |
| BMK_Unigene_56368 | 1.32E-07 | 7.061805967 | PREDICTED: chymotrypsin-2-like [Musca domestica] |  |
| BMK_Unigene_02946 | 8.89E-20 | 6.704877948 | PREDICTED: trypsin beta-like [Zeugodacus cucurbitae] |  |
| BMK_Unigene_01107 | 1.58E-06 | 6.318079972 | PREDICTED: putative leucine-rich repeat-containing protein DDB_G0290503 [Bactrocera dorsalis] |  |
| BMK_Unigene_61379 | 3.18E-13 | 5.083638928 | hypothetical protein FF38_00502 [Lucilia cuprina] |  |
| BMK_Unigene_57881 | 7.05E-06 | 4.350323819 | PREDICTED: lysyl oxidase homolog 2A [Bactrocera oleae] |  |
| BMK_Unigene_64331 | 4.05E-08 | 3.95301315 | PREDICTED: vanin-like protein 2 [Musca domestica] |  |
| BMK_Unigene_56670 | 1.14E-06 | 3.425323637 | PREDICTED: uncharacterized protein LOC105226199 [Bactrocera dorsalis] |  |
| BMK_Unigene_66063 | 5.22E-07 | 3.130677009 | PREDICTED: putative fatty acyl-CoA reductase CG5065 [Zeugodacus cucurbitae] |  |
| LS |  |  |  | |
| BMK_Unigene_04046 | 1.19E-17 | 7.802456793 | PREDICTED: uncharacterized protein LOC108376199 [Rhagoletis zephyria] | |
| BMK_Unigene_03889 | 0.000234609 | 5.674651938 | hypothetical protein SAMD00019534_048350 [Acytostelium subglobosum LB1] | |
| BMK_Unigene_07983 | 5.78E-10 | 3.977319562 | PREDICTED: keratin, type II cytoskeletal 1b [Zeugodacus cucurbitae] | |
| BMK_Unigene_16111 | 0.000895715 | 3.436261046 | PREDICTED: uncharacterized protein LOC108131964 [Drosophila bipectinata] | |
| BMK_Unigene_18245 | 7.24E-17 | 2.942047028 | PREDICTED: uncharacterized protein LOC108361514 [Rhagoletis zephyria] | |
| BMK_Unigene_08755 | 0.002153 | 2.689210432 | glycine-rich cell wall structural protein 1.8-like [Lucilia cuprina] | |
| BMK_Unigene_56684 | 4.09E-47 | 2.615537969 | PREDICTED: bumetanide-sensitive sodium-(potassium)-chloride cotransporter [Bactrocera dorsalis] | |
| BMK_Unigene_14730 | 3.09E-05 | 2.567950637 | uncharacterized protein LOC105664721 [Ceratitis capitata] | |
| BMK_Unigene_54898 | 4.99E-08 | 2.556823622 | uncharacterized protein LOC105664721 [Ceratitis capitata] | |
| BMK_Unigene_21479 | 1.51E-06 | 2.436020005 | pol polyprotein, partial [Drosophila buzzatii] | |
