# Supplementary material for: Comparative transcriptome analysis of Liriomyza trifolii (Burgess) and Liriomyza sativae (Blanchard) (Diptera: Agromyzidae) in response to rapid cold hardening
Source: PLoS One. 2022 Dec 15;17(12):e0279254. doi: 10.1371/journal.pone.0279254 (PMC9754249; doi:10.1371/journal.pone.0279254)
Supplement: S2 Table — (DOCX) [file pone.0279254.s008.docx]

**S2 Table**. Upregulated (log2), annotated DEGs in CS vs RCH of both species

| #ID | FDR | Log_2_FC | NR Annotation |
| --- | --- | --- | --- |
| **Lt** |  |  |  |
| BMK_Unigene_00913 | 0.00298649 | 1.349674016 | centrosomal protein of 164 kDa [Ceratitis capitata] |
| BMK_Unigene_13026 | 0.024016748 | 1.282720141 | probable cytochrome P450 303a1 [Lucilia cuprina] |
| BMK_Unigene_70387 | 0.017048183 | 1.220328902 | uncharacterized protein Dyak_GE27401 [Drosophila yakuba] |
| BMK_Unigene_61326 | 0.011148039 | 1.066663883 | PREDICTED: uncharacterized protein LOC108033051 [Drosophila biarmipes] |
| BMK_Unigene_10011 | 0.009342115 | 1.054045536 | uncharacterized protein Dpse_GA20083, isoform A [Drosophila pseudoobscura pseudoobscura] |
| BMK_Unigene_54956 | 0.045989189 | 1.014359171 | PREDICTED: uncharacterized histidine-rich protein DDB_G0274557 [Stomoxys calcitrans] |
| BMK_Unigene_67546 | 0.02578517 | 1.011974408 | PREDICTED: solute carrier family 22 member 13 [Drosophila kikkawai] |
| **Ls** |  |  | centrosomal protein of 164 kDa [Ceratitis capitata] |
| BMK_Unigene_14730 | 4.46E-14 | 1.875403791 | uncharacterized protein LOC105664721 [Ceratitis capitata] |
| BMK_Unigene_54898 | 3.89E-06 | 1.869954291 | uncharacterized protein LOC105664721 [Ceratitis capitata] |
| BMK_Unigene_10625 | 1.02E-15 | 1.757874771 | PREDICTED: uncharacterized protein LOC108033051 [Drosophila biarmipes] |
| BMK_Unigene_10027 | 0.000175587 | 1.699351559 | apolipoprotein D-like isoform X2 [Parasteatoda tepidariorum] |
| BMK_Unigene_56030 | 0.000141826 | 1.547736676 | thioredoxin-T [Drosophila willistoni] |
| BMK_Unigene_11804 | 8.88E-05 | 1.543100778 | PREDICTED: protein nutcracker isoform X1 [Musca domestica] |
| BMK_Unigene_66297 | 0.000665468 | 1.507077637 | PREDICTED: PDZ domain-containing protein GIPC3 [Bactrocera latifrons] |
| BMK_Unigene_17250 | 7.85E-14 | 1.431175548 | GH18058 [Drosophila grimshawi] |
| BMK_Unigene_00968 | 2.49E-09 | 1.376843486 | PREDICTED: LOW QUALITY PROTEIN: serine protease easter [Bactrocera oleae] |
| BMK_Unigene_60478 | 0.000281834 | 1.375008325 | serine, glycine and glutamine-rich protein [Drosophila serrata] |
