# Supplementary material for: Comparative transcriptome analysis of Liriomyza trifolii (Burgess) and Liriomyza sativae (Blanchard) (Diptera: Agromyzidae) in response to rapid cold hardening
Source: PLoS One. 2022 Dec 15;17(12):e0279254. doi: 10.1371/journal.pone.0279254 (PMC9754249; doi:10.1371/journal.pone.0279254)
Supplement: S3 Table — (DOCX) [file pone.0279254.s009.docx]

**S3 Table.** Common upregulated (log2) and down regulation of annotated DEGs in Control vs RCH and CS vs RCHCS.

| #ID | NR annotation |
| --- | --- |
| **Control vs RCH** |  |
| **UP** |  |
| BMK_Unigene_67546 | PREDICTED: solute carrier family 22 member 13 [Drosophila kikkawai] |
| BMK_Unigene_00968 | PREDICTED: LOW QUALITY PROTEIN: serine protease easter [Bactrocera oleae] |
| BMK_Unigene_42226 | PREDICTED: endocuticle structural protein SgAbd-6 [Bactrocera dorsalis] |
| BMK_Unigene_13026 | probable cytochrome P450 303a1 [Lucilia cuprina] |
| BMK_Unigene_04446 | PREDICTED: alpha-protein kinase 1 [Drosophila busckii] |
| **DOWN** |  |
| BMK_Unigene_15205 | PREDICTED: cytochrome P450 4c3, partial [Rhagoletis zephyria] |
| BMK_Unigene_04052 | histidine-rich glycoprotein isoform X2 [Ceratitis capitata] |
| BMK_Unigene_01043 | PREDICTED: glucose dehydrogenase [FAD, quinone]-like [Musca domestica] |
| BMK_Unigene_04428 | PREDICTED: mastermind-like protein 2 isoform X2 [Drosophila bipectinata] |
| BMK_Unigene_63406 | elongation of very long chain fatty acids protein 7 isoform X1 [Ceratitis capitata] |
| **CS vs RCHCS** |  |
| **UP** |  |
| BMK_Unigene_67546 | PREDICTED: solute carrier family 22 member 13 [Drosophila kikkawai] |
| BMK_Unigene_54956 | PREDICTED: uncharacterized histidine-rich protein DDB_G0274557 [Stomoxys calcitrans] |
| BMK_Unigene_10011 | uncharacterized protein Dpse_GA20083, isoform A [Drosophila pseudoobscura pseudoobscura] |
| **DOWN** |  |
| BMK_Unigene_16880 | Uncharacterized protein Dana_GF10533 [Drosophila ananassae] |
| BMK_Unigene_04428 | PREDICTED: mastermind-like protein 2 isoform X2 [Drosophila bipectinata] |
| BMK_Unigene_02777 | PREDICTED: uncharacterized protein LOC108603899 [Drosophila busckii] |
| BMK_Unigene_61487 | uncharacterized protein LOC111684248 isoform X2 [Lucilia cuprina] |
| BMK_Unigene_66069 | Cationic amino acid transporter 2 [Lucilia cuprina] |
| BMK_Unigene_15205 | PREDICTED: cytochrome P450 4c3, partial [Rhagoletis zephyria] |
| BMK_Unigene_01043 | PREDICTED: glucose dehydrogenase [FAD, quinone]-like [Musca domestica] |
